# Improved Method for Estimating Reaction Rates During Push‐Pull Tests

**DOI:** 10.1111/gwat.12770

**Published:** 2018-04-30

**Authors:** Charles J. Paradis, Emma R. Dixon, Lauren M. Lui, Adam P. Arkin, Jack C. Parker, Jonathan D. Istok, Edmund Perfect, Larry D. McKay, Terry C. Hazen

**Affiliations:** ^1^ Department of Earth and Planetary Sciences University of Tennessee Knoxville TN; ^2^ Biosciences Division Oak Ridge National Laboratory Oak Ridge TN; ^3^ Department of Civil and Environmental Engineering University of Tennessee Knoxville TN; ^4^ Environmental Genomics and Systems Biology Division Lawrence Berkeley National Laboratory Berkeley CA; ^5^ Department of Bioengineering University of California Berkeley CA; ^6^ School of Civil and Construction Engineering Oregon State University Corvallis OR; ^7^ Department of Microbiology University of Tennessee Knoxville TN; ^8^ Center for Environmental Biotechnology University of Tennessee Knoxville TN; ^9^ Institute for a Secure and Sustainable Environment University of Tennessee Knoxville TN

## Abstract

The breakthrough curve obtained from a single‐well push‐pull test can be adjusted to account for dilution of the injection fluid in the aquifer fluid. The dilution‐adjusted breakthrough curve can be analyzed to estimate the reaction rate of a solute. The conventional dilution‐adjusted method assumes that the ratios of the concentrations of the nonreactive and reactive solutes in the injection fluid vs. the aquifer fluid are equal. If this assumption is invalid, the conventional method will generate inaccurate breakthrough curves and may lead to erroneous conclusions regarding the reactivity of a solute. In this study, a new method that generates a dilution‐adjusted breakthrough curve was theoretically developed to account for any possible combination of nonreactive and reactive solute concentrations in the injection and aquifer fluids. The newly developed method was applied to a field‐based data set and was shown to generate more accurate dilution‐adjusted breakthrough curves. The improved dilution‐adjusted method presented here is simple, makes no assumptions regarding the concentrations of the nonreactive and reactive solutes in the injection and aquifer fluids, and easily allows for estimating reaction rates during push‐pull tests.

## Abstract 2

*Article impact statement*: A new method was developed to generate dilution‐adjusted breakthrough curves from push‐pull tests to more accurately estimate reaction rates.

## Introduction

The push‐pull test is a powerful site characterization method and has been applied in a wide range of hydrological settings including saturated and unsaturated soils, sediments and surface water bodies (Istok 2013). Push‐pull tests are particularly useful for estimating reaction rates of solutes (Haggerty et al. 1998; Snodgrass and Kitanidis 1998). In a groundwater setting, a push‐pull test is conducted by injecting a volume of water containing one or more nonreactive and reactive solutes into a single well (push phase), followed by a nonpumping period (drift phase), and subsequent extraction of groundwater from the same well (pull phase). The extracted groundwater is a mixture of the injection and aquifer fluids. The concentration of the reactive solute in the extraction fluid can be adjusted for dilution to generate a concentration vs. time‐elapsed profile (dilution‐adjusted breakthrough curve) as given by Istok (2013, equation 4.23):

(1) $$C_{e}^{2*}=C_{e}^{2}\left[\frac{C_{i}^{1}}{C_{e}^{1}}\right]$$

where $C_{i}^{1}$is the concentration of nonreactive solute in the injection fluid (M/L^3^), $C_{e}^{1}$ is the concentration of nonreactive solute in the extraction fluid (M/L^3^), $C_{e}^{2}$ is the concentration of reactive solute in the extraction fluid (M/L^3^) and $C_{e}^{2*}$ is the dilution‐adjusted concentration of reactive solute in the extraction fluid (M/L^3^).

Analysis of Equation 1 shows that the concentration of the reactive solute in the extraction fluid $\left(C_{e}^{2}\right)$ is multiplied by the inverse of the relative change in the concentration of the nonreactive solute in the extraction vs. the injection fluid $\left(C_{i}^{1}/C_{e}^{1}\right)$ to yield a dilution‐adjusted concentration of the reactive solute in the extraction fluid $\left(C_{e}^{2*}\right)$. For example, if the concentration of the nonreactive solute in the extraction fluid $\left(C_{e}^{1}\right)$ decreased twofold with respect to its injected concentration $\left(C_{i}^{1}\right)$ then the concentration of the reactive solute in the extraction fluid $\left(C_{e}^{2}\right)$ is multiplied twofold to generate a dilution‐adjusted concentration $\left(C_{e}^{2*}\right)$.

The conventional dilution‐adjusted method, as shown in Equation 1, assumes that the mass transport characteristics of both the nonreactive and reactive solutes, for example, advection, mechanical dispersion, molecular diffusion, and sorption, are not different. As a result, the conventional dilution‐adjusted method dictates that any deviation of the dilution‐adjusted breakthrough curve from its injected concentration can be attributed to reactivity. It is important to note that the mass transport characteristics of both the nonreactive and reactive solutes must be well understood in the context of the groundwater setting. The reaction rate of a solute can be estimated by fitting an appropriate kinetic model, for example, zero‐order, first‐order, Michaelis‐Menton, etc., to the dilution‐adjusted breakthrough curve (Istok 2013).

The conventional dilution‐adjusted method also assumes that the ratios of the concentrations of the nonreactive and reactive solutes in the injection fluid vs. the aquifer fluid are equal as given by:

(2) $$\begin{array}{c} \frac{C_{i}^{1}}{C_{a}^{1}}=\frac{C_{i}^{2}}{C_{a}^{2}} \end{array}$$

where$\;C_{a}^{1}$ is the concentration of nonreactive solute in the aquifer fluid (M/L^3^), $C_{i}^{2}$is the concentration of reactive solute in the injection fluid (M/L^3^)$\;\text{and}\;C_{a}^{2}$ is the concentration of reactive solute in the aquifer fluid (M/L^3^).

For example, if the injected concentration of the nonreactive solute $\left(C_{i}^{1}\right)$ is 100 times greater than its aquifer concentration $\left(C_{a}^{1}\right)$ this would yield a maximum dilution‐adjusted factor of 100 for the reactive solute. However, if the injected concentration of the reactive solute $\left(C_{i}^{2}\right)$ is only 10 times greater than its aquifer concentration $\left(C_{a}^{2}\right)$ then a maximum, and physically correct, dilution‐adjusted factor would only be 10. In the case presented here, the conventional dilution‐adjusted method would generate a breakthrough curve which over adjusts for dilution. Therefore, the conventional dilution‐adjusted method has the potential to generate invalid breakthrough curves that can lead to erroneous conclusions regarding the reactivity of a solute. Presumably, in the many previously published studies that utilized the conventional dilution‐adjusted method to estimate reaction rates, the assumptions associated with Equation 1, most notably those shown in Equation 2, were either valid, or adjustments were made to those assumptions during data analysis to achieve a reasonable level of validity (Istok 2013). However, no study to date has clearly established a dilution‐adjusted method to account for the likely scenario where the ratios of the concentrations of the nonreactive and reactive solutes in the injection fluid vs. the aquifer fluid are not equal.

The objectives of this study were the following: (1) theoretically develop a dilution‐adjusted method that generates the breakthrough curve of a reactive solute when the ratios of the concentrations of the nonreactive and reactive solutes in the injection fluid vs. the aquifer fluid are not equal and (2) apply and compare the newly developed method with the conventional dilution‐adjusted method using a field‐based data set from a previously published study.

## Conventional Dilution‐Adjusted Method: Valid and Invalid Examples

The conventional dilution‐adjusted method will generate valid breakthrough curves when the ratios of the concentrations of the nonreactive and reactive solutes in the injection fluid vs. the aquifer fluid are equal as given by Equation 2. For example 1 of Table 1, suppose that bromide and ethanol are added to the injection fluid as nonreactive and reactive solutes, respectively, and both at concentrations 100 times greater than in the aquifer fluid.

**Table 1:** Theoretical Example Numbers 1 through 3 of Push‐Pull Test Parameters for Bromide (Br^−^), a Nonreactive Solute, and for Ethanol (EtOH), a Reactive Solute

	Br^−^			EtOH			
Example No.	$C_{i}^{1}$ (mg/L)	$C_{a}^{1}$ (mg/L)	$k_{d}^{1}$ (1/h)	$C_{i}^{2}$ (mg/L)	$C_{a}^{2}$ (mg/L)	$k_{d}^{2}\;\left(1/h\right)$	$k_{r}^{2}$ (1/h)
Example 1	100	1	−0.2	50	0.5	−0.2	−0.2
Example 2	100	1	−0.2	50	0.5	−0.2	0.0
Example 3	100	1	−0.2	50	5	−0.2	0.0

$C_{i}^{1}$ is the concentration of nonreactive solute in the injection fluid, $C_{a}^{1}$ is the concentration of nonreactive solute in the aquifer fluid, $k_{d}^{1}$ is the dilution rate of nonreactive solute, $C_{i}^{2}$is the concentration of reactive solute in the injection fluid, $C_{a}^{2}$is the concentration of reactive solute in the aquifer fluid, $k_{d}^{2}$ is the dilution rate of reactive solute and $k_{r}^{2}$ is the reaction rate of reactive solute.

Bromide is commonly utilized as a nonreactive solute due to its conservative behavior in a wide range of groundwater settings (Davis et al. 1980). Ethanol is frequently utilized as a reactive solute to stimulate microbial‐mediated remediation of a wide range of groundwater contaminants (Weier et al. 1994; Istok et al. 2004; Hrapovic et al. 2005; Wu et al. 2006; Rodriguez‐Freire et al. 2016). Nonreactive and reactive solutes are typically injected at concentrations that greatly exceed those in the aquifer fluid to yield sufficiently high signal to noise ratios (Istok 2013). Suppose, further that the dilution of the injection fluid and the reaction of ethanol obey first‐order kinetics, with rate constants of *k*_d_ and *k*_r_, respectively (Table 1). Dilution and reaction rates are typically modeled using zero‐, first‐, or Michaelis‐Menton kinetics (Istok et al. 1997; Haggerty et al. 1998; Snodgrass and Kitanidis 1998; Hageman et al. 2003; Yang et al. 2007). For example 1, the dilution rates of bromide and ethanol are equal (−0.2/h) but ethanol has a reaction rate (−0.2/h) (Table 1). Finally, suppose that the pull phase begins immediately after the push phase, that is, no drift phase. Therefore, the concentration of bromide is equal to its injected concentration at the time equal to 0 which corresponds to the start of the pull phase.

**Figure 1:**
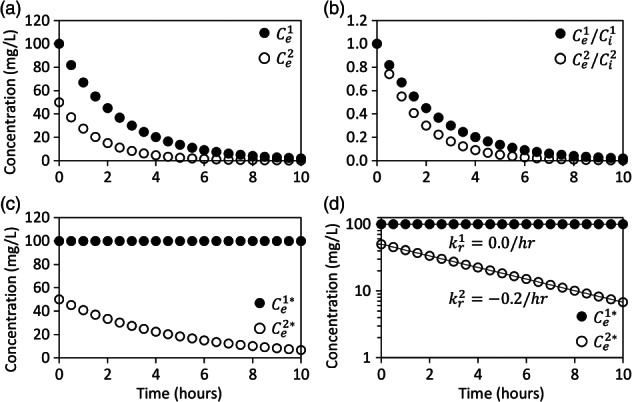
Example breakthrough curves of bromide (closed circle), a nonreactive solute, and ethanol (open circle), a reactive solute, in the extraction fluid for example 1 (Table 1) that are: not adjusted for dilution (a), relative to their injected concentrations (b), adjusted for dilution (c), and adjusted for dilution on a semi‐logarithmic plot (d). $C_{e}^{1}$ is the concentration of nonreactive solute in the extraction fluid, $C_{e}^{2}$ is the concentration of reactive solute in the extraction fluid, $C_{i}^{1}$ is the concentration of nonreactive solute in the injection fluid, $C_{i}^{2}$ is the concentration of reactive solute in the injection fluid, $C_{e}^{1*}$ is the dilution‐adjusted concentration of nonreactive solute in the extraction fluid, $C_{e}^{2*}$ is the dilution‐adjusted concentration of reactive solute in the extraction fluid, $k_{r}^{1}$ is the first‐order reaction rate of nonreactive solute and $k_{r}^{2}$ is the first‐order reaction rate of reactive solute.

The nonadjusted breakthrough curves of bromide and ethanol in the extraction fluid exponentially decrease from their injected concentrations and approach their aquifer concentrations (Figure 1a). However, it is not possible to readily assess the effect of dilution on the breakthrough curve of ethanol (Figure 1a). A plot of the relative breakthrough curves of bromide and ethanol, that is, the concentration in the extraction fluid divided by the concentration in the injection fluid, clearly show that the breakthrough of ethanol is visibly less than the breakthrough of bromide (Figure 1b). The relative breakthrough curves suggest that dilution alone cannot account for the breakthrough of ethanol and that a reaction occurred (Figure 1b). The dilution‐adjusted breakthrough curves of bromide and ethanol, from Equation 1, show that the concentration of bromide is always equal to its injected concentration whereas the concentration of ethanol exponentially decreases from its injected concentration (Figure 1c). It is known a priori that the dilution and reactivity of ethanol obey first‐order kinetics (Table 1), and therefore, a semi‐logarithmic plot of the dilution‐adjusted breakthrough curves should follow a straight line (Figure 1d). Exponential regression of the semi‐logarithmic plot of the dilution‐adjusted breakthrough curves yield reaction rates of 0.0 and −0.2/h for bromide and ethanol, respectively (Figure 1d). Therefore, Equation 1 allowed for estimating the reaction rate (−0.2/h) of ethanol. The net mass of ethanol removal can be quantified by integrating the area under the dilution‐adjusted concentration vs. volume extracted profile and subtracting from it the injected concentration multiplied by the extracted volume.

**Figure 2:**
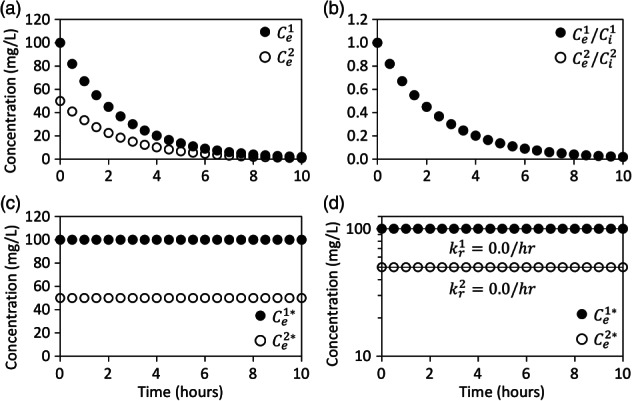
Example breakthrough curves of bromide (closed circle), a nonreactive solute, and ethanol (open circle), a reactive solute, in the extraction fluid for example 2 (Table 1) that are: not adjusted for dilution (a), relative to their injected concentrations (b), adjusted for dilution (c), and adjusted for dilution on a semi‐logarithmic plot (d). $C_{e}^{1}$ is the concentration of nonreactive solute in the extraction fluid, $C_{e}^{2}$ is the concentration of reactive solute in the extraction fluid, $C_{i}^{1}$ is the concentration of nonreactive solute in the injection fluid, $C_{i}^{2}$ is the concentration of reactive solute in the injection fluid, $C_{e}^{1*}$ is the dilution‐adjusted concentration of nonreactive solute in the extraction fluid, $C_{e}^{2*}$ is dilution‐adjusted concentration of reactive solute in the extraction fluid, $k_{r}^{1}$ is the first‐order reaction rate of nonreactive solute and $k_{r}^{2}$ is the first‐order reaction rate of reactive solute.

For example 2, suppose again that bromide and ethanol are added to the injection fluid at concentrations 100 times greater than in the aquifer fluid but that ethanol has no reaction rate (Table 1). The reactivity of ethanol can be negligible upon first exposure and increase upon subsequent exposures to groundwater settings (Kline et al. 2011). The nonadjusted breakthrough curves of bromide and ethanol in the extraction fluid exponentially decrease from their injected concentrations and approach their aquifer concentrations (Figure 2a). A plot of the relative breakthrough curves clearly show that the breakthrough of ethanol is identical to the breakthrough of bromide (Figure 2b). The relative breakthrough curves suggest that dilution alone can account for the breakthrough of ethanol and that no reaction occurred (Figure 2b). The dilution‐adjusted breakthrough curves show that the concentrations of bromide and ethanol are always equal to their injected concentrations (Figure 2c). Exponential regression of the semi‐logarithmic plot of the dilution‐adjusted breakthrough curves reaction rates of 0.0 and 0.0/h for bromide and ethanol, respectively (Figure 2d).

**Figure 3:**
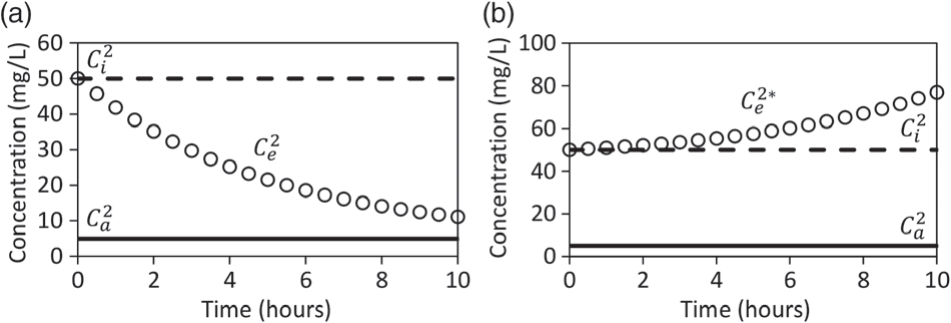
Example breakthrough curves of ethanol (open circle), a reactive solute, for example 3 (Table 1) that are: not adjusted for dilution (a) and adjusted for dilution (b), the bold dashed line$:C_{i}^{2}$ is the concentration of ethanol in the injection fluid, the bold solid line:$\;C_{a}^{2}$ is the concentration of ethanol in the aquifer fluid, $C_{e}^{2}$ is the concentration of ethanol in the extraction fluid, $C_{e}^{2*}$ is the dilution‐adjusted concentration of ethanol in the extraction fluid.

The utility of Equation 1 for estimating reaction rates has been demonstrated in numerous studies (Istok 2013). However, it must be re‐emphasized that Equation 1 assumes that the ratios of the concentrations of the nonreactive and reactive solutes in the injection fluid vs. the aquifer fluid are equal as given by Equation 2. For examples 1 and 2, the ratios of the concentrations of the nonreactive and reactive solutes in the injection fluid vs. the aquifer fluid were both equal to 100 (Table 1). Suppose for example 3 that the ratios of the concentrations of the nonreactive and reactive solutes in the injection fluid vs. the aquifer fluid were not equal because the concentration of ethanol in the aquifer fluid was increased to 5 mg/L whereas all other push‐pull tests parameters were identical to example 2, that is, ethanol and bromide have identical dilution rates and neither has a reaction rate (Table 1). The nonadjusted breakthrough curve of ethanol exponentially decreases from its injected concentration and approaches its aquifer concentration (Figure 3a). The dilution‐adjusted breakthrough curve of ethanol shows that its concentration exponentially increases above its injected concentration (Figure 3b), and this is physically incorrect. The conventional dilution‐adjusted method generated a breakthrough curve of ethanol for example 3 that over adjusted for dilution because Equation 2 was invalid. An accurate dilution‐adjusted breakthrough curve of ethanol for example 3 would be identical to its injected concentration. Therefore, if the assumptions in Equation 2 are invalid, as was the case for example 3 (Table 1), then Equation 1 will be invalid and may lead to erroneous conclusions regarding the reactivity of a solute.

## New Dilution‐Adjusted Method: Theoretical Development

During the push phase of a push‐pull test, a finite volume of fluid (*V*_i_) that contains a known mass of a nonreactive solute (*M*_i_) is injected into an aquifer. The aquifer consists of an infinite volume of fluid that contains a known concentration of the nonreactive solute (*C*_a_). During the pull phase, the extraction fluid (*C*_e_) is periodically sampled over time (*t*) as given by:

(3) $$\begin{array}{c} C_{e}=f\left(V_{i},M_{i},C_{a},t\right) \end{array}$$

where *V*_*i*_ is the volume of injection fluid (L^3^), *M*_*i*_ is the mass of nonreactive solute in the injection fluid (M), *C*_a_ is the concentration of nonreactive solute in the aquifer fluid (M/L^3^), *C*_e_ is the concentration of nonreactive solute in the extraction fluid (M/L^3^) and *t* is the time elapsed from beginning of pull phase (*T*).

Equation 3 can be simplified as:

(4) $$\begin{array}{c} C_{e}=f\left(C_{i},C_{a},t\right) \end{array}$$

where *C*_*i*_ is the concentration of nonreactive solute in the injection fluid or *M*_i_ divided by *V*_i_ (M/L^3^). The concentration of the nonreactive solute in the extraction fluid (*C*_e_) will approach that of the aquifer (*C*_a_) as time (*t*) approaches infinity as given by:

(5) $$\begin{array}{c} \lim_{t\to \infty }C_{e}\left(C_{i},C_{a},t\right)=C_{a} \end{array}$$

The limit shown in Equation 5 assumes that *C*_i_ and *C*_a_ are constants and that only *C*_e_ and *t* are variables. If the concentration of the nonreactive solute in the injection fluid (*C*_i_) is either greater than or less than the concentration of the nonreactive solute in the aquifer fluid (*C*_a_) and if the pull phase begins immediately after the push phase, that is, no drift phase, Equation 5 can be described as either a decreasing or increasing function that approaches the concentration of the aquifer fluid (*C*_a_). The initial condition at time equal to 0 for *C*_e_ is given by:

(6) $$\begin{array}{c} C_{e}\left(t=0\right)=C_{i} \end{array}$$

The final condition as time approaches infinity for *C*_e_ is given by:

(7) $$\begin{array}{c} C_{e}\left(t\to \infty \right)=C_{a} \end{array}$$

The limit shown in Equation 5 can be calculated using a modified first‐order kinetics model that satisfies the initial and final conditions in 6 and 7, respectively, as given by:

(8) $$\begin{array}{c} C_{e}\left(t\right)=\left[C_{i}-C_{a}\right]e^{-\mathrm{kt}}+C_{a} \end{array}$$

where *k* is the dilution rate 1/[*T*] and is greater than 0.

It is important to note that many solutions can be found that obey the limit shown in Equation 5 and satisfy the initial and final conditions in 6 and 7, respectively, for example, nonlinear solutions, piecewise linear solutions, or converging sequences. The modified first‐order kinetics model is presented here for simplicity and relevancy. Analysis of Equation 8 at time equal to 0 yields *C*_e_ equal to *C*_i_ and as time approaches infinity yields *C*_e_ equal to *C*_a_. Equation 8 can be rearranged as follows:

(9) $$\frac{\left[C_{e}\left(t\right)-C_{a}\right]}{\left[C_{i}-C_{a}\right]}\begin{array}{c} =e^{-\mathrm{kt}} \end{array}$$

Analysis of Equation 9 shows that the ratio of the numerator to the denominator ranges from 1 at time equal to 0, to 0 as time approaches infinity. For Equation 9, a value of 1 indicates no dilution, a value of 0 indicates complete dilution, and values between 1 and 0 indicate partial dilution. The term on the right‐hand side of Equation 9 describes the behavior of dilution. In the case presented here, the behavior of dilution is nonlinear and can be described using a modified first‐order kinetic model. If the behavior of dilution of the nonreactive and reactive solutes is the same, and if it obeys the limit shown in Equation 5, and if it satisfies the initial and final conditions in 6 and 7, respectively, then Equation 9 can be written as:

(10) $$\frac{\left[C_{e}^{1}-C_{a}^{1}\right]}{\left[C_{i}^{1}-C_{a}^{1}\right]}=\frac{\left[C_{e}^{2e}-C_{a}^{2}\right]}{\left[C_{i}^{2}-C_{a}^{2}\right]}$$

where $C_{e}^{2e}$ is the expected concentration of reactive solute in the extraction fluid (M/L^3^).

Equation 10 can be rearranged to solve for the expected concentration of a reactive solute in the extraction fluid as given by:

(11) $$\begin{array}{c} C_{e}^{2e}=\left(\frac{\left[C_{e}^{1}-C_{a}^{1}\right]}{\left[C_{i}^{1}-C_{a}^{1}\right]}\right)\left[C_{i}^{2}-C_{a}^{2}\right]+C_{a}^{2} \end{array}$$

Equation 11 generates the expected concentration of a reactive solute in the extraction fluid due to dilution between the injection and aquifer fluids. Equation 11 assumes the following: (1) the concentrations of both solutes are equal to their injection concentrations at time equal to 0, (2) the concentrations of both solutes are equal to their aquifer concentrations as time approaches infinity, and (3) the mass transport characteristics of both solutes, for example, advection, mechanical dispersion, molecular diffusion, and sorption, are not different.

During a push‐pull test, each independent variable in Equation 11 is measured. Therefore, Equation 11 can be used to compare the expected concentration of a reactive solute in the extraction fluid $(C_{e}^{2e}$) to the measured concentration of a reactive solute in the extraction fluid $(C_{e}^{2}$). Any difference between the two concentrations can be attributed to reactivity when assuming the mass transport characteristics of both solutes, for example, advection, mechanical dispersion, molecular diffusion, and sorption, are not different. Equation 11, unlike Equation 1, makes no assumptions regarding the ratios of the concentrations of the nonreactive and reactive solutes in the injection fluid vs. the aquifer fluid. Rather, Equation 11 accounts for such differences and allows for a direct comparison of the expected vs. measured breakthrough curves. Finally, Equation 11 can be utilized to generate a dilution‐adjusted breakthrough curve of a reactive solute in the extraction fluid as given by:

(12) $$\begin{array}{c} C_{e}^{2**}=C_{e}^{2}\left[\frac{C_{i}^{2}}{C_{e}^{2e}}\right] \end{array}$$

where $C_{e}^{2**}$ is the dilution‐adjusted concentration of reactive solute in the extraction fluid (M/L^3^).

Analysis of Equation 12 shows that when the measured $\left(C_{e}^{2}\right)$ and expected $\left(C_{e}^{2e}\right)$ concentrations of the reactive solute are equal, the dilution‐adjusted concentration $\left(C_{e}^{2**}\right)$ is equal to the injected concentration $\left(C_{i}^{2}\right)$. Likewise, when the measured $\left(C_{e}^{2}\right)$ and expected $\left(C_{e}^{2e}\right)$ concentrations are not equal, the dilution‐adjusted concentration $\left(C_{e}^{2**}\right)$ is either greater than or less than the injected concentration. Therefore, analysis of the dilution‐adjusted breakthrough curve from Equation 12, unlike Equation 1, can be utilized to estimate the reaction rate of a solute for any possible combination of nonreactive and reactive solute concentrations in the injection and aquifer fluids.

## New Dilution‐Adjusted Method: Field‐Based Application

The new dilution‐adjusted method presented here (Equation 12) was applied to a previously published study by Paradis et al. (2016), and the results were compared to those from the conventional dilution‐adjusted method (Equation 1). Paradis et al. (2016) utilized the push‐pull test method to investigate the mobility of reduced and immobilized uranium in the presence of nitrate oxidant and analyzed the data using the conventional dilution‐adjusted method (Equation 1). Paradis et al. (2016) concluded that reduced sulfur‐bearing species, as opposed to reduced uranium‐bearing species, were preferentially oxidized and mobilized. This conclusion was based on the following: (1) analyzing the magnitudes and trends of the dilution‐adjusted breakthrough curves of nitrate, nitrite, sulfate, and uranium and (2) quantifying the mass of uranium and sulfate recovered during the pull phase relative to bromide, that is, recovery factors. Recovery factors greater than 1 indicated that more uranium or sulfate was recovered relative to bromide. Recovery factors less than 1 indicated that less sulfate or uranium was recovered relative to bromide.

In the Paradis et al. (2016) study, bromide and nitrate were added as nonreactive and reactive solutes, respectively, to a 200‐L injection fluid at concentrations much greater than in the aquifer fluid (Table 2). The injection fluid also contained uranium at a concentration much greater than in the aquifer fluid (Table 2). The concentrations of chloride, nitrite, and sulfate in the injection fluid were only slightly greater than in the aquifer fluid (Table 2). The 200‐L fluid was injected by siphon into a test well constructed in a shallow, unconfined groundwater setting primarily comprised of reworked fill materials. The drift phase was negligible because groundwater was periodically extracted from the test well the following day and continued for 36 days. The push‐pull tests were conducted simultaneously in a set of three wells (FW219, FW220, FW225). Only the breakthrough curves for test well FW220 are presented here for brevity. However, the zero‐order reaction rates and recovery factors for all three test wells are presented here for completeness.

**Table 2:** Concentrations of Nonreactive (Br^−^, Cl^−^) and Reactive Solutes (NO_3_^−^, NO_2_^−^, SO_4_^2−^, U(VI)) in the Injection and Aquifer Fluids of Test Well FW220 from Paradis et al. (2016)

Fluid	Br^−^ (mM)	Cl^−^ (mM)	NO_3_^−^ (mM)	NO_2_^−^ (mM)	SO_4_^2−^ (mM)	U(VI) (μM)
Injection	0.52	0.43	93.8	0.0024	1.0	5.4
Aquifer	0.000068	0.17	0.127	0.00036	0.3	0.2
Inj./Aq.	7631	2.6	739	6.7	3.1	30

Inj./Aq. is the ratio of the solute concentration in the injection vs. aquifer fluid.

**Figure 4:**
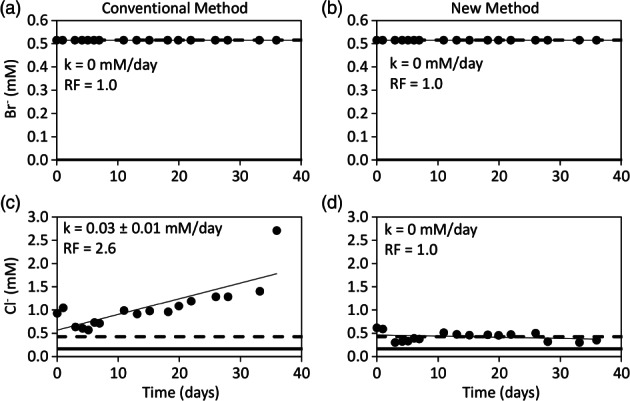
Dilution‐adjusted breakthrough curves of bromide (Br^−^) and chloride (Cl^−^) of test well FW220 from the conventional method (a), (c) and the new method (b), (d). Bold solid line represents the aquifer concentration, bold dashed line represents the injected concentration, thin solid line represents the linear regression of the dilution‐adjusted data, *k* is the zero‐order reaction rate plus or minus its 95% confidence interval and RF is the recovery factor.

The conventional and new dilution‐adjusted breakthrough curves of bromide were identical to their injected concentrations (Figure 4a and 4b). The zero‐order reaction rates were 0 and the recovery factors were 1 (Figure 4a and 4b). Identical results for bromide were observed in the two other test wells (Tables 3 and 4). These results were expected because Equations 1 and 12 are equivalent when generating the dilution‐adjusted breakthrough curves of the pre‐defined nonreactive solute, for example, bromide. If the dilution‐adjusted breakthrough curve of the pre‐defined nonreactive solute is not identical to its injected concentration then one or more assumptions of the method used to generate the breakthrough curve is invalid. The conventional dilution‐adjusted breakthrough curve of chloride was notably greater than the injected concentration (Figure 4c). The zero‐order reaction rate was 0.03 ± 0.01 mM/day and the recovery factor was 2.6 (Figure 4c). In contrast, the new dilution‐adjusted breakthrough curve of chloride was nearly identical to the injected concentration (Figure 4d). The zero‐order reaction rate was 0 and the recovery factor was 1 (Figure 4d). Similar results for chloride were observed in the two other test wells (Tables 3 and 4). When considering that chloride has been shown to be a nonreactive solute in a wide range of groundwater settings (Davis et al. 1980), including the study site (Hu and Moran 2005), it seems unlikely that a reaction would occur as indicated by the conventional dilution‐adjusted method (Figure 4c). Rather, no reaction would be expected for chloride as shown by the new dilution‐adjusted method (Figure 4d). Therefore, it is likely that the new method provided a more accurate breakthrough curve of chloride than the conventional method. A probable explanation for the failure of the conventional method to generate a breakthrough curve of chloride nearly identical to the injection concentration is that the ratio of the concentration of the bromide and chloride in the injection fluid vs. the aquifer fluid were far from equal (Table 1). Therefore, the assumption in Equation 2 was clearly invalid which strongly suggests that Equation 1 was invalid for the case of chloride.

**Table 3:** Zero‐Order Rates of Nonreactive (Br^−^, Cl^−^) and Reactive (NO_3_^−^, NO_2_^−^, SO_4_^2−^, U(VI)) Solutes from the Conventional and New Dilution‐Adjusted Methods

	Conventional Method						New Method					
Well ID	Br^−^	Cl^−^	NO_3_^−^	NO_2_^−^	SO_4_^2−^	U(VI)	Br^−^	Cl^−^	NO_3_^−^	NO_2_^−^	SO_4_^2−^	U(VI)
219	0	0.03	−1.3	0	0.63	0.62	0	−0.003	−1.0	0	0	0.13
220	0	0.03	−3.2	−0.05	0.60	0.31	0	0	−3.2	−0.03	0.11	0.18
225	0	0.09	−3.2	−0.04	0.61	0.53	0	0	−3.2	−0.01	0	0
Average	0	0.05	−2.6	−0.03	0.62	0.49	0	−0.001	−2.5	−0.01	0.04	0.11
S.E.	0	0.02	0.6	0.01	0.01	0.09	0	0.001	0.7	0.01	0.04	0.05

Units of rates are mM/day for all solutes except U(VI), units for U(VI) are μM/day, rates equal to 0 are not significant (*p* > 0.05), rates not equal to 0 are significant (*p* < 0.05), S.E. is the standard error.

**Table 4:** Recovery Factors of Nonreactive (Br^−^, Cl^−^) and Reactive (NO_3_^−^, NO_2_^−^, SO_4_^2−^, U(VI)) Solutes from the Conventional and New Dilution‐Adjusted Methods

	Conventional Method						New Method					
Well ID	Br^−^	Cl^−^	NO_3_^−^	NO_2_^−^	SO_4_^2−^	U(VI)	Br^−^	Cl^−^	NO_3_^−^	NO_2_^−^	SO_4_^2−^	U(VI)
219	1.0	2.9	0.1	1902	24	2.4	1.0	0.2	0.1	2.7	2.5	1.0
220	1.0	2.6	0.3	302	13	1.9	1.0	1.0	0.3	219	5.2	1.6
225	1.0	5.9	0.2	44	22	2.4	1.0	0.8	0.2	5.4	4.8	0.9
Average	1.0	3.8	0.2	749	20	2.2	1.0	0.7	0.2	76	4.1	1.2
S.E.	0.0	1.0	0.05	581	3.6	0.2	0.0	0.2	0.05	71	0.8	0.2

Recovery factors greater than 1 indicate a net production, recovery factors less than 1 indicate a net removal, recovery factors equal to 1 indicate a no net change, S.E. is the standard error.

**Figure 5:**
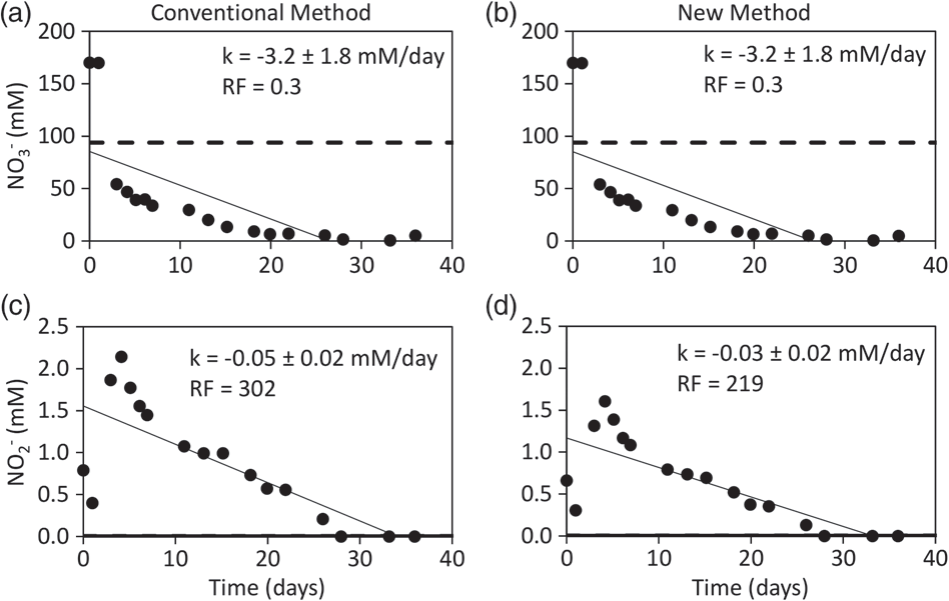
Dilution‐adjusted breakthrough curves of nitrate (NO_3_^−^) and nitrite (NO_2_^−^) of test well FW220 from the conventional method (a), (c) and the new method (b), (d). Bold solid line represents the aquifer concentration, bold dashed line represents the injected concentration, thin solid line represents the linear regression of the dilution‐adjusted data, *k* is the zero‐order reaction rate plus or minus its 95% confidence interval and RF is the recovery factor.

The conventional and new dilution‐adjusted breakthrough curves of nitrate were practically identical (Figure 5a and 5b). The zero‐order reaction rates were −3.2 ± 1.8 mM/day and the recovery factors were 0.3 (Figure 5a and 5b). Both breakthrough curves of nitrite were very similar (Figure 5c and 5d). The zero‐order reaction rates were −0.05 ± 0.02 and −0.03 ± 0.02 mM/day, respectively (Figure 5c and 5d). The recovery factors were 302 and 219, respectively (Figure 5a and 5b). Similar results for nitrate and nitrite were observed in the two other test wells (Tables 3 and 4). Nitrate reduction to nitrite and other reduced nitrogen‐bearing species was expected to occur. Therefore, it is likely that nitrate removal was concomitant with nitrite production as indicated by both methods (Figure 5, Tables 3 and 4).

The practically identical breakthrough curves of nitrate from both methods (Figure 5a and 5b) were expected because both bromide and nitrate were added to the injection fluid at concentrations that greatly exceeded those in the aquifer fluid (Table 2). Therefore, Equation 1, that assumes that the ratio of the concentrations of the nonreactive and reactive solutes in the injection fluid vs. the aquifer fluid are equal, was likely valid. The very similar breakthrough curves of nitrite from both methods (Figure 5c and 5d) were somewhat surprising because nitrite, like chloride, was not added to the injection fluid and its concentration only slightly exceeded that in the aquifer fluid (Table 2). A probable explanation for both methods to generate very similar breakthrough curves of nitrite is that its extracted concentrations greatly exceeded both those in the injection and aquifer fluids (data not shown). Therefore, neither the injection nor the aquifer fluid likely had a notable dilution effect on the breakthrough curves of nitrite. It should be noted that it may not be necessary to adjust the extracted concentration of a reactionary product when its injected and aquifer concentrations are similar and its extracted concentrations are relatively high (Istok 2013), as for the case of nitrite. However, applying the new dilution‐adjusted method that accounts for any possible combination of nonreactive and reactive solute concentrations in the injection and aquifer fluids to all extracted solutes will allow for a direct comparison of all breakthrough curves and subsequent analyses. It should also be noted that linear regression of the dilution‐adjusted breakthrough of nitrite is not ideal for predictive purposes because nitrite is typically an intermediate product of nitrate reduction followed by reduction to other nitrogen‐bearing species.

**Figure 6:**
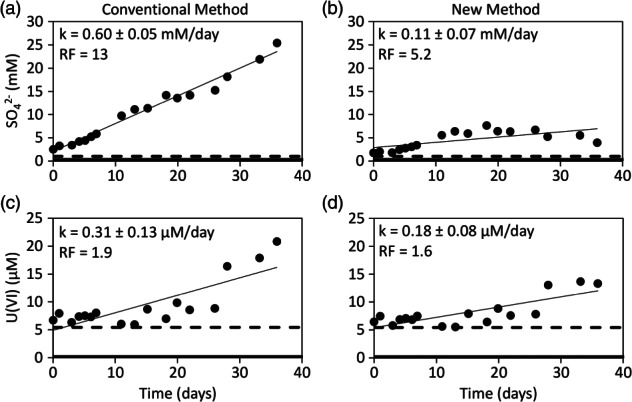
Dilution‐adjusted breakthrough curves of sulfate (SO_4_^2−^) and uranium (U(VI)) of test well FW220 from the conventional method (a), (c) and the new method (b), (d). Bold solid line represents the aquifer concentration, bold dashed line represents the injected concentration, thin solid line represents the linear regression of the dilution‐adjusted data, *k* is the zero‐order reaction rate plus or minus its 95% confidence interval and RF is the recovery factor.

The conventional and new dilution‐adjusted breakthrough curves of sulfate were not similar (Figure 6a and 6b). The conventional dilution‐adjusted breakthrough curve of sulfate showed a strong and near‐linear increase from approximately 3 to 25 mM with a zero‐order reaction rate of 0.60 ± 0.05 mM/day and a recovery factor of 13 (Figure 6a). In contrast, the new dilution‐adjusted breakthrough curve of sulfate showed a slight and somewhat linear increase from approximately 3 to 6 mM with a zero‐order reaction rate of 0.11 ± 0.07 mM/day and a recovery factor of 5.2 (Figure 6b). Similar results for sulfate were observed in the two other test wells (Tables 3 and 4). Although sulfate production was expected to occur, the rate and extent of sulfate production from the conventional method was notably greater than from the new method (Tables 3 and 4). The dissimilar breakthrough curves, reaction rates, and recovery factors of sulfate from both methods (Figure 6a and 6b, Tables 3 and 4) was somewhat expected because the extracted concentrations of sulfate, unlike nitrite, did not greatly exceed both those in the injection and aquifer fluids (data not shown). Therefore, the injection and aquifer fluid likely had a notable effect on the breakthrough curve of sulfate. It is likely that the new dilution‐adjusted breakthrough curve of sulfate, as opposed to the conventional, was more accurate because it accounted for any possible combination of nonreactive and reactive solute concentrations in the injection and aquifer fluids.

The conventional and new dilution‐adjusted breakthrough curves of uranium were fairly similar (Figure 6c and 6d). The zero‐order reaction rates were 0.31 ± 0.13 and 0.18 ± 0.08 mM/day, respectively (Figure 6c and 6d). The recovery factors were 1.9 and 1.6, respectively (Figure 6c and 6d). Fairly similar results for uranium were observed in the two other test wells (Tables 3 and 4). Although some uranium production was expected to occur, the rate and extent of uranium production from the conventional method was slightly greater than from the new method (Tables 3 and 4). Nevertheless, both methods suggested that the rate and extent of uranium production was substantially less than sulfate (Tables 3 and 4). The fairly similar breakthrough curves, reaction rates, and recovery factors of uranium from both methods (Figure 6c and 6d, Tables 3 and 4) was somewhat expected because uranium, like bromide, was present in the injection fluid at a concentration that greatly exceeded that in the aquifer fluid (Table 2). Therefore, Equation 1, which assumes that the ratio of the concentrations of the nonreactive and reactive solutes in the injection fluid vs. the aquifer fluid are equal was likely valid.

In summary, the conventional and new dilution‐adjusted methods produced practically identical zero‐order reaction rates and recovery factors for the solutes added to the injection fluid at concentrations much greater than in the aquifer fluid, for example, bromide and nitrate (Tables 3 and 4). In contrast, the conventional method produced exaggerated rates and recovery factors, as compared to the new method, for the solutes not added to the injection fluid, for example, chloride, nitrite, sulfate, and uranium (Tables 3 and 4). A likely explanation for the conventional method to exaggerate the rates and recovery factors of nonadded solutes is that it can overestimate, and subsequently over adjust, the effect of dilution between the injection and aquifer fluids.

## Conclusions

The conventional method used to generate dilution‐adjusted breakthrough curves during push‐pull tests was shown to be invalid when the ratio of the concentrations of the nonreactive and reactive solutes in the injection fluid vs. the aquifer fluid are not equal. A new dilution‐adjusted method was theoretically developed to account for any possible combination of nonreactive and reactive solute concentrations in the injection and aquifer fluids. The utility of the newly developed method was demonstrated by applying it to a field‐based data set. The newly developed method was shown to be advantageous relative to the conventional method by generating less exaggerated and subsequently more realistic dilution‐adjusted breakthrough curves. The improved dilution‐adjusted method presented here is simple and easily allows for estimating reaction rates of solutes during push‐pull tests.

It is important to note that the improved method, like the conventional method, assumes that the mass transport characteristics of both the nonreactive and reactive solutes, for example, advection, mechanical dispersion, molecular diffusion, and sorption, are not different and that any deviation of the dilution‐adjusted breakthrough curve from its injected concentration can be attributed to reactivity. However, the improved method can potentially be applied to better characterize mass transport characteristics if multiple nonreactive solutes are utilized. For example, diffusive mass transport can be characterized by analyzing the dilution‐adjusted breakthrough curves of multiple nonreactive solutes with differing aqueous diffusion coefficients. Any deviation of the dilution‐adjusted breakthrough curve from its injected concentration can be attributed to diffusive mass transport if advection, mechanical dispersion, and sorption are no different among the multiple nonreactive solutes. Likewise, sorption can be characterized by analyzing the dilution‐adjusted breakthrough curves of multiple nonreactive solutes with differing sorption behavior. Any deviation of the dilution‐adjusted breakthrough curve from its injected concentration can be attributed to sorption if advection, mechanical dispersion, and molecular diffusion are no different among the multiple nonreactive solutes. Analysis of the dilution‐adjusted breakthrough curves for characterizing diffusive mass transport or sorption would likely require an analytical or numerical transport model, with or without diffusion‐controlled exchange between mobile and immobile pore water, respectively.

## Authors' Note

The author(s) does not have any conflicts of interest or financial disclosures to report.
